# The dynamic history of prokaryotic phyla: discovery, diversity and division

**DOI:** 10.1099/ijsem.0.006508

**Published:** 2024-09-09

**Authors:** Mark J. Pallen

**Affiliations:** 1 Norwich Medical School, University of East Anglia, Norwich Research Park, Norwich, Norfolk, UK; 2 Quadram Institute Bioscience, Norwich Research Park, Norwich, Norfolk, UK

**Keywords:** *Candidatus* names, phylogenetics, prokaryotic nomenclature, prokaryotic phyla, prokaryotic taxonomy, shotgun metagenomics

## Abstract

Here, I review the dynamic history of prokaryotic phyla. Following leads set by Darwin, Haeckel and Woese, the concept of phylum has evolved from a group sharing common phenotypes to a set of organisms sharing a common ancestry, with modern taxonomy based on phylogenetic classifications drawn from macromolecular sequences. Phyla came as surprising latecomers to the formalities of prokaryotic nomenclature in 2021. Since then names have been validly published for 46 prokaryotic phyla, replacing some established names with neologisms, prompting criticism and debate within the scientific community. Molecular barcoding enabled phylogenetic analysis of microbial ecosystems without cultivation, leading to the identification of candidate divisions (or phyla) from diverse environments. The introduction of metagenome-assembled genomes marked a significant advance in identifying and classifying uncultured microbial phyla. The lumper–splitter dichotomy has led to disagreements, with experts cautioning against the pressure to create a profusion of new phyla and prominent databases adopting a conservative stance. The *Candidatus* designation has been widely used to provide provisional status to uncultured prokaryotic taxa, with phyla named under this convention now clearly surpassing those with validly published names. The Genome Taxonomy Database (GTDB) has offered a stable, standardized prokaryotic taxonomy with normalized taxonomic ranks, which has led to both lumping and splitting of pre-existing phyla. The GTDB framework introduced unwieldy alphanumeric placeholder labels, prompting recent publication of over 100 user-friendly Latinate names for unnamed prokaryotic phyla. Most candidate phyla remain ‘known unknowns’, with limited knowledge of their genomic diversity, ecological roles, or environments. Whether phyla still reflect significant evolutionary and ecological partitions across prokaryotic life remains an area of active debate. However, phyla remain of practical importance for microbiome analyses, particularly in clinical research. Despite potential diminishing returns in discovery of biodiversity, prokaryotic phyla offer extensive research opportunities for microbiologists for the foreseeable future.

## Historical introduction

*‘*I believe that the arrangement of the groups within each class, in due subordination and relation to the other groups, must be strictly genealogical in order to be natural*…*’ Charles Darwin, 1859 [1].

*‘*A revolution is occurring in biology…The cell is basically an historical document, and gaining the capacity to read it (by the sequencing of genes) cannot but drastically alter the way we look at all of biology. No discipline within biology will be more changed by this revolution than microbiology*…’* Carl Woese, 1987 [2].

There is something fundamental about prokaryotic phyla—deep branches on the great tree of life, early stopping points *en route* from root to tip. Yet they come with a mixed track record within microbiology, inspiring awe through discovery and diversity, while provoking division among academics as to how they should be classified. They also represent surprising latecomers to the formalities of prokaryotic nomenclature. Plus, as prokaryotic phyla now number in the hundreds, there are reasons for questioning how useful it is to continue to view them as basic units of taxonomy, evolution or ecology.

The term *phylum* was coined by the German naturalist Ernst Haeckel in an 1866 monograph on the morphology of organisms, as a Latinized form of *phylon*, itself a transliteration of the ancient Greek word *ϕῦλον*, meaning race or tribe [3]. The term *phylum* has since been used widely in zoology to denote a group of animals sharing a body plan because of descent from a common ancestor. At least since de Candolle’s 1868 *Laws of Botanical Nomenclature* [4], botanists have tended to prefer the term *division*, although the International Code of Nomenclature for algae, fungi, and plants now accepts the terms *division* and *phylum* as equivalent [5].

Although Linnaeus laid the foundations of modern taxonomy [6], it was Charles Darwin who recast classification as an exercise in genealogy (or as we would now say, phylogenetics) [1]. Darwin acolyte Ernst Haeckel produced ornate evolutionary trees placing micro-organisms in the kingdom *Protista* and putting what we now call bacteria into the phylum *Moneres* (later called *Monera*) [3]. By the 1870s, Ferdinand Cohn had demarcated four ‘tribes’ of bacteria: *Sphaerobacteria* (spherical bacteria), *Microbacteria* (rod-shaped bacteria), *Desmobacteria* (filamentous bacteria) and *Spirobacteria* (spiral bacteria) [7].

Modern viewpoints on the highest-ranking bacterial taxa date back to the work of Canadian microbiologist Robert G.E. Murray and his colleagues, who proposed—informally in 1962 and then formally in 1978—that bacteria should be divided into three divisions on the basis of Gram-stain and cell-wall structure [8,9]: *Gracilicutes* (‘thin skins’; for Gram-negative cells, including cyanobacteria), *Firmacutes* (‘firm skins’; for Gram-positive cells; later corrected to *Firmicutes*) and *Mollicutes* (‘soft skins’; for those lacking cell walls). *Gracilicutes* and *Firmacutes* were subsequently included in the *Approved List of Bacterial Names* [10].

In the 1970s, Carl Woese and George Fox took the pioneering first steps towards a phylogenetic classification of prokaryotes using rRNA sequences [11]. They proposed ‘three aboriginal lines of descent’ for cellular life: the ‘eubacteria’ (typical bacteria; later becoming the domain *Bacteria* [12]), the ‘archaebacteria’ (represented by methanogens; later becoming the domain Archaea [12]) and the ‘urkaryotes’ (representing the cytoplasmic component of eukaryotic cells). At first, Woese recognized just three major subdivisions among bacteria: the Gram-positives, the Gram-negatives and the blue-green-algae-plus-chloroplasts [11]. He saw mycoplasmas as merely ‘degenerate’ clostridia [13], which led him to demote the *Mollicutes* to a sub-branch of the Gram-positive phylum.

By the mid-1980s, Woese and his colleagues had gathered evidence to support the existence of at least 10 bacterial and four archaeal phyla [2,14, 15]. In his landmark review on bacterial evolution, Woese boasted ‘the definition of the eubacterial ‘Phyla’ or ‘Divisions’… brings our understanding of bacterial systematics on a par with eukaryotic systematics’ [2].

A surprising discovery within the new evolutionary paradigm was that few bacterial phyla could be defined by phenotypic properties common to all members of a group. Thus, Woese noted that while all spirochaetes possess characteristic axial fibrils, some members of the ‘Gram-positive’ phylum lack Gram-positive cell walls and there was no convincing phenotypic circumscription for what he called the bacteroides–flavobacteria phylum (now the *Bacteroidota*). As Woese saw it, ‘such groups challenge the microbiologist to discover their unifying phenotypic motifs’ [2].

In the second edition of *Bergey’s Manual of Systematic Bacteriology*, published in 2001, Editor-in-Chief George Garrity and his colleagues followed Woese in unequivocally adopting a phylogenetic taxonomy, based on rRNA sequences rather than phenotypes to define new taxa. The Bergey’s Manual team embraced the term *phylum*, rather than *division* and used the term to describe 23 named phyla within Bacteria and two phyla within Archaea. Descriptions names for prokaryotic phyla with cultured representatives have accumulated slowly but steadily since then, so that there are now over forty named phyla with cultured representatives (Table 1). However, as prokaryotic nomenclature and taxonomy remain separate endeavours, not all phyla with validly published names are universally accepted as warranting this rank.

**Table 1.** Prokaryotic phyla with validly published names (as of November 2023)

Phylum name	Synonyms	Valid publication	Description
**Domain Archaea**			
Methanobacteriota, Halobacteriota, Thermoplasmatota*Note that these names can be considered validly published heterotypic synonyms or non-synonymous if adopting a taxonomic opinion where the nomenclatural type is not assigned to another phylum	*Halobacterota*, *Euryarchaeota*	[28,103]	[104,105]
*Nanobdellota*	*Nanoarchaeota*	[28]	[106]
*Nitrososphaerota*	*Thaumarchaeota*	[27]	[107]
*Thermoproteota*	*Crenarchaeota*	[27]	[108]
**Domain Bacteria**			
*Abditibacteriota*	Candidate Division FBP	[28]	[109]
*Acidobacteriota*	*Acidobacteria*	[27]	[110]
*Actinomycetota*	*Actinobacteraeota*, *Actinobacteria*, *Actinobacteriotaa*	[27]	[36]
*Aquificota*	*Aquificae*, *Aquificaeota*	[27]	[111]
*Armatimonadota*	*Armatimonadetes*, *Armatimonadaeota*, *OP10*	[27]	[112]
*Atribacterota*	*Candidatus* Atribacteria, Candidate Divisions OP9 and JS1	[27]	[113]
*Bacillota*	*Firmacutes*, *Firmacutes*	[27]	[9]
*Bacteroidota*	*Bacteroidetes*	[27]	[114]
*Balneolota*	*Balneolaeota*	[27]	[115]
*Bdellovibrionota*		[27]	[86]
*Caldisericota*	*Caldiserica*, *Caldisericaeota*, Candidate Division OP5	[27]	[116]
*Calditrichota*	*Calditrichaeota*	[27]	[117]
*Campylobacterota*	*Epsilonbacteraeota*	[27]	[85]
*Chlamydiota*	*Chlamydiae*, *Chlamydaeota*	[27]	[118]
*Chlorobiota*	*Chlorobi*; GTDB considers this a class within *Bacteroidota*	[27]	[119]
*Chloroflexota*	*Chloroflexi*, *Chloroflexaeota*	[27]	[120]
*Chrysiogenota*	*Chrysiogenetes*, *Chrysiogenaeota*, *Chrysiogenetota*	[27]	[121]
*Coprothermobacterota*		[27]	[122]
*Cyanobacteriota*	*Cyanobacteria*	[123]	[123]
*Deferribacterota*	*Deferribacteres*	[27]	[124]
*Deinococcota*	*Deinococcus–Thermus*	[27]	[125]
Desulfobacterota	LPSN considers this a synonym of *Pseudomonadota*	[28]	[86]
*Dictyoglomerota*	*Dictyoglomi*, *Dictyoglomota*	[27]	[126]
*Elusimicrobiota*	*Elusimicrobia*, Candidate Division OP7, Termite Group 1 (TG1)	[27]	[127]
*Fibrobacterota*	*Fibrobacteres*	[27]	[118]
*Fusobacteriota*	*Fusobacteria*	[27]	[118]
*Gemmatimonadota*	*Gemmatimonadetes*, Candidate Division BD	[27]	[128]
*Ignavibacteriota*	*Ignavibacteriae*, Candidate Division ZB1;	[27]	[129]
*Kiritimatiellota*	*Kiritimatiellaeota*	[27]	[130]
*Lentisphaerota*	*Lentisphaerae*	[27]	[131]
*Mycoplasmatota*	*Tenericutes*	[27]	[132]
*Myxococcota*		[27]	[86]
*Nitrospinota*	*Nitrospinae*	[27]	[133]
*Nitrospirota*	*Nitrospirae*	[27]	[134]
*Planctomycetota*	*Planctomycetes*	[27]	[118]
*Pseudomonadota*	*Proteobacteria*	[27]	[135]
*Rhodothermota*	*Rhodothermaeota*	[27]	[136]
*Spirochaetota*	*Spirochaetes*	[27]	[118]
*Synergistota*	*Synergistetes*	[27]	[137]
*Thermodesulfobacteriota*	*Thermodesulfobacteria*	[27]	[138]
*Thermomicrobiota*	*Thermomicrobia*	[27]	[139]
*Thermosulfidibacterota*	LPSN considers this a synonym of *Aquificota*	[103]	[103]
*Thermotogota*	*Thermotogae,* Candidate Division OP2, Candidate Division EM3	[27]	[140]
*Verrucomicrobiota*	*Verrucomicrobia*	[27]	[141]

* *Methanobacteriota,Halobacteriota* and *Thermoplasmatota* can be considered heterotypic synonyms, validly published under the ICNPInternational Code of Nomenclature of Prokaryotes. Alternatively, *each* is a correct name instead if this phylum is regarded as a separate phylum (i.e., if its nomenclatural type is not assigned to another phylum whose name is validly published, legitimate and not rejected and has priority).

## Naming prokaryotic phyla

Reflecting the botanical roots of bacterial taxonomy, the 1947*,* 1958 and 1966 bacterial codes all proposed the term *division* (in Latin *divisio*) for the highest rank in the classification of bacteria, although they failed to provide any examples of named divisions [16,18]. For reasons that are unclear, the rank of division was dropped from the 1975 revision to the Code and remained absent from the subsequent revisions in 1990 and 2008 [19,21]. Names for two divisions named by Gibbons and Murray in 1978—*Firmacutes* and *Gracilicutes*—are recorded in the 1980 *Approved Lists of Bacterial Names*. However, responses to a recent request for opinion have clarified that this does not grant them standing in nomenclature [22,23]. In fact, the absence of the rank of phylum from the Code (until recently, see below) means that none of the phylum names generated over recent decades have any standing, however widely used.

The earliest attempts to create well-formed Latin names for prokaryotic phyla adopted varied approaches. In some cases, microbiologists created descriptive names without a precise link to an existing genus name, e.g. *Firmicutes*, *Tenericutes*, *Proteobacteria* and *Actinobacteria*. However, in most cases, the phylum name was simply the nominative plural form of the name of a representative genus. Examples from the first declension include *Chlamydiae*, *Nitrospirae*, *Lentisphaerae* and *Thermotogae*; those from the second declension include *Acidobacteria*, *Chloroflexi*, *Elusimicrobia* and *Fusobacteria*; while those from the third declension include *Fibrobacteres*, *Planctomycetes* and *Spirochaetes*. However, the supposed plural forms were not always formed grammatically: for example, the correct versions of *Aquificae*, *Armatimonadetes* and *Bacteroidetes* should be *Aquifices*, *Armatimonades* and *Bacteroides*.

In 2015, a multinational team launched a proposal to include the rank of phylum within the International Code of Nomenclature of Prokaryotes (ICNP), so that phylum names could obtain standing in nomenclature [24]. Rather than adopt descriptive names or eponyms for phyla, they suggested adding the arbitrary suffix *-aeota* to the stem of a relevant class to generate the phylum names. By 2018, a consensus had emerged in favour of the simpler ending *-ota* [25]. The International Committee on Systematics of Prokaryotes (ICSP) voted on the issue in February 2021, favouring *-ota* over *-aeota* and genus over class as nomenclatural type for phylum names [26]. Most committee members also voted in favour of allowing the Judicial Commission to make exceptions to the use of the chosen suffix, although this flexibility has never been invoked.

Shortly afterwards, nomenclature expert Aharon Oren and colleagues validly published names for 46 prokaryotic phyla with cultured type strains [27,28], mostly presented as corrections of effectively published synonyms (Table 1). Most new names showed a resemblance to existing names, e.g. *Bacteroidota* replacing *Bacteroidetes* or *Spirochaetota* replacing *Spirochaetes*. However, some well-established descriptive names were replaced by completely new names built from bacterial genera, e.g. *Pseudomonadota* replacing *Proteobacteria*, *Bacillota* replacing *Firmicutes*, *Crenarchaeota* replacing *Thermoproteota* and *Thaumarchaeota* replacing *Nitrososphaerota*.

Overwriting names that had been used for decades generated criticism [29,33]— which drew responses from the ICSP [34,35]. Nonetheless major online resources such as Genome Taxonomy Database (GTDB) and the National Center for Biotechnology Information (NCBI) have now adopted the new names.

Painting new names on to old phyla raises an intriguing issue at the intersection between taxonomy and nomenclature. The earliest phylum names, such as *Firmicutes* and *Proteobacteria*, are associated with taxonomic definitions and circumscriptions now universally seen as outmoded. Thus, *Firmicutes* was defined by Gibbons and Murray in 1978 as ‘prokaryotes having a Gram-positive type of cell wall’ and as far as they were concerned included the order *Actinomycetales* [9]. However, for over a decade, the order *Actinomycetales* has been assigned by most authorities to a distinct phylum now called *Actinomycetota* [36]. Thus replacement of the term *Firmicutes* by *Bacillota* serves as a reminder that the taxonomy associated with the former name has been extensively revised. Similar arguments apply to the replacement of *Proteobacteria* with *Pseudomonadota*. However, when validly publishing phylum names, Oren and Garrity simply referred to the original definition of each phylum [27]. Now that a type genus has been assigned to each phylum name, it should be fairly straightforward to publish new genome-basic descriptions and circumscriptions in line with current practice.

## Accommodating the uncultured majority

In the 1990s, amplification of molecular bar codes paved the way for phylogenetic analysis of microbial ecosystems that did away with the requirement for cultivation [37]. Descriptions soon emerged of ‘candidate divisions’ (or ‘candidate phyla’) including:

- the *OS-K group*, recovered from Octopus Spring in Yellowstone National Park [38].
- the *Marine Group A*, recovered from the Pacific Ocean [39] (now termed *Candidatus* Neomarinimicrobiota)
- the *Termite Group 1*, recovered from the termite gut [40] (now termed *Elusimicrobiota*).

Pioneering studies in the late 1990s by Philip Hugenholtz, Norman Pace and others on sequences from environmental sources revealed astonishing microbial diversity. A single hot spring in Yellowstone National Park—the Obsidian Pool—provided evidence for 12 new candidate divisions (OP1-OP12), where a candidate division was defined as ‘an unaffiliated lineage in multiple analyses… having <85% identity to reported sequences’ [41]. A contaminated aquifer at an abandoned air force base in Michigan yielded another six candidate divisions, WS1–WS6 [42]. In a 1998 review article, Hugenholtz and colleagues reported that, over the previous decade, the number of identifiable bacterial divisions had more than tripled to around 40 [43]. Five years later, Rappé and Giovannoni [44], when reporting on 52 bacterial phyla, included 26 candidate phyla that contained no known cultivated representatives (Table 2).

**Table 2.** Prokaryotic *Candidatus* phyla with type genera (as of November 2023)

Phylum name	Synonyms
**Domain Archaea**	
*Candidatus* Aenigmatarchaeota	Aenigmarchaeota
*Candidatus* Altarchaeota	Altiarchaeota
*Candidatus* Baldrarchaeota	
*Candidatus* Borrarchaeota	
*Candidatus* Freyrarchaeota	
*Candidatus* Geothermarchaeota	THSCG (Terrestrial Hot Springs Crenarchaeotic Group)
*Candidatus* Hadarchaeota	
*Candidatus* Hermodarchaeota	
*Candidatus* Hodarchaeota	
*Candidatus* Huberarchaeota	Huberarchaea
*Candidatus* Hydrothermarchaeota	Marine benthic group E
*Candidatus* Iainarchaeota	Diapherotrites
*Candidatus* Kariarchaeota	
*Candidatus* Korarchaeota	
*Candidatus* Lokiarchaeota	Lokiarchaeia
*Candidatus* Micrarchaeota	
*Candidatus* Nanohalarchaeota	
*Candidatus* Njordarchaeota	
*Candidatus* Odinarchaeota	
*Candidatus* Parvarchaeota	
*Candidatus* Poseidoniota	Thermoplasmatota, Diaforarchaea group
*Candidatus* Sifarchaeota	
*Candidatus* Sigynarchaeota	
*Candidatus* Undinarchaeota	
*Candidatus* Wukongarchaeota	
**Domain Bacteria**	
*Candidatus* Acetithermota	Acetothermia; potentially heterotypic synonym of Bipolaricaulota
*Candidatus* Aerophobota	Aerophobetes, Candidate Division CD12, Candidate Division BHI80-139
*Candidatus* Altimarinota	Gracilibacteria, Candidate Division GN02
*Candidatus* Aminicenantota	Aminicenantes, Candidate Division OP8
*Candidatus* Babelota	Dependentiae, TM6
*Candidatus* Binatota	Candidate Division UBP10
*Candidatus* Bipolaricaulota	Potentially heterotypic synonym of Acetothermia; Candidate Division OP1
*Candidatus* Caldatribacteriota	
*Candidatus* Caldipriscota	Pyropristinus
*Candidatus* Calescibacteriota	Calescamantes, Candidate Division EM19
*Candidatus* Canglongiota	
*Candidatus* Cloacimonadota	Cloacimonetes, Candidate Division WWE1
*Candidatus* Cryosericota	
*Candidatus* Deferrimicrobiota	Candidate Phylum MBNT15
*Candidatus* Dormiibacterota	Dormibacteraeota, Candidate Division AD3
*Candidatus* Electryoneota	Candidate Division AABM5-125-24; arbitrary *Candidatus* name Debefiota [87]
*Candidatus* Elulimicrobiota	Elulota
*Candidatus* Eremiobacterota	Eremiobacteraeota, Candidate Division WPS-2, Palusbacterota
*Candidatus* Fermentibacterota	Fermentibacteria, Candidate Division Hyd24-12
*Candidatus* Fervidibacterota	Fervidibacteria, Candidate Division OctSpa1-106
*Candidatus* Heilongiota	
*Candidatus* Hinthialibacterota	Arbitrary *Candidatus* name Urusiota [87], GTDB designation p_OLB16
*Candidatus* Hydrogenedentota	Hydrogenedentes, Candidate Division NKB19
*Candidatus* Hydrothermota	Hydrothermae, Candidate Division EM 3, Candidate Division WOR-3,
*Candidatus* Kapaibacteriota	Kapabacteria, Kapaibacteria
*Candidatus* Krumholzibacteriota	
*Candidatus* Kryptoniota	Kryptonia
*Candidatus* Latescibacterota	Latescibacteria
*Candidatus* Lernaellota	Arbitrary *Candidatus* name Podoxiota [87], GTDB designation p_FEN-1099
*Candidatus* Macinerneyibacteriota	Mcinerneyibacteriota
*Candidatus* Methanomethylicota	Verstraetearchaeota
*Candidatus* Microgenomatota	Microgenomates, Candidate Division OP11
*Candidatus* Moduliflexota	Modulibacteria
*Candidatus* Muiribacteriota	Muirbacteria
*Candidatus* Neomarinimicrobiota	Marinimicrobia, SAR406 cluster, marine group A
*Candidatus* Omnitrophota	Omnitrophica, Candidate Divisions OP3, WOR-2
*Candidatus* Paceibacterota	Pacebacteria
*Candidatus* Parcunitrobacterota	Parcunitrobacteria
*Candidatus* Qinglongiota	
*Candidatus* Rifleibacteriota	Riflebacteria
*Candidatus* Saccharimonadota	Saccharibacteria, Candidate Division TM7
*Candidatus* Sumerlaeota	Candidate Division BRC1
*Candidatus* Tianyaibacteriota	Tianyabacteria
*Candidatus* Wirthibacterota	Wirthbacteria

A key step forward for uncultured phyla came from the recovery of metagenome-assembled genomes (MAGs) and use of conserved protein-coding genes as phylogenetic markers [45,48]. Steady improvements in sequencing and bioinformatics [49] culminated in genomic documentation of over a dozen candidate phyla by 2013 [50,60]. Improvements in single-cell genome sequencing led to Rinke and colleagues to report on new candidate bacterial phyla hiding within ‘microbial dark matter’ [61]. Crucially, Rinke *et al*. assigned Latin names (with protologues and type species) to 14 candidate bacterial phyla, two candidate bacterial superphyla (Patescibacteria and Terribacteria) and four candidate archaeal phyla.

## Splitters versus lumpers

As long ago as 1857, Charles Darwin claimed ‘it is good to have hair-splitters and lumpers’ [62]. American palaeontologist George Gaylord Simpson popularized the terms ‘lumpers’ and ‘splitters’ in a 1945 book on the principles of classification [63], The lumper–splitter dichotomy emerged for prokaryotic phyla with a provocative proposal in 2014 for a uniform classification of cultured and uncultured prokaryotes, which defined a phylum with a cut-off of ≤75% sequence identity in the 16S rRNA gene [64]. Unapologetic splitters, the authors proclaimed, ‘Taxonomists might have been too cautious and tended to classify new taxa into existing units… rather than proposing new ones’. They proposed that 15 recognized candidate phyla should be split into 83 new candidate phyla, dividing a single candidate phylum OD1—which by then had been named *Candidatus* Parcubacteria—into 28 new phyla. They also claimed that the silva database of 16S rRNA gene sequences was home to as many as 1500 prokaryotic phyla. This proposal was not accepted by the wider community.

Proliferation of proposed prokaryotic phyla began again in 2015 following metagenomic analysis of an aquifer near Rifle, Colorado by Banfield and her team. After analysing over 800 MAGs, they defined a new high-level bacterial lineage, the Candidate Phyla Radiation (CPR) [65]. Within this lineage, they elevated the candidate phyla Microgenomates (OP11) and Parcubacteria (OD1) to the rank of superphyla and then proceeded to delineate and name 26 new candidate phyla within them. The following year, Banfield and her collaborators proposed another 47 new phyla, after analysing thousands of MAGS from the same aquifer [66].

This zeal for splitting has meant that, within a decade, the Banfield team named nearly 100 candidate bacterial phyla without designating type genera (Table 3) [65,78]. However, the status of these groups as phylum-level taxa has not been met with universal approval. For example, in a 2017 review of rRNA gene reference databases, Glöckner *et al*. [79] wrote ‘The numbers of bacterial and archaeal phyla are currently under a dramatic expansion… While most studies employ a reasonable phylogenetic reconstruction strategy to propose new phyla, some maybe a result of the enormous pressure on scientists to create the deepest taxonomic rank possible, rendering their work more dramatic… As such, we employ a conservative measure of not including every single new phylum in the silva taxonomic framework’. At the time of writing only a handful of the phyla named by the Banfield team appear in the commonly used silva or GTDB taxonomies or appear when browsing the rank of phylum in the List of Prokaryotic names with Standing in Nomenclature (LPSN) [80,81].

**Table 3.** Prokaryotic phyla listed without type genera (as of November 2023)

Published name	Oren and Göker name	Name proposed here	References
**Domain Archaea**			
*Candidatus* Aigarchaeota	*Candidatus* Augarchaeota		[51]
*Candidatus* Asgardarchaeota	*Candidatus* Asgardarchaeota		[142]
*Candidatus* Bathyarchaeota			[143]
*Candidatus* Brockarchaea	*Candidatus* Brockiarchaeota		[144]
*Candidatus* Geoarchaeota			[145]
*Candidatus* Gerdarchaeota			[146]
*Candidatus* Hadesarchaeota			[147]
*Candidatus* Heimdallarchaeia	*Candidatus* Heimdallarchaeota		[148,149]
*Candidatus* Helarchaeota			[150]
*Candidatus* Marsarchaea	*Candidatus* Martarchaeota		[151]
*Candidatus* Nezhaarchaeota			[152]
*Candidatus* Pacearchaeota			[153]
*Candidatus* Thorarchaeia	*Candidatus* Thorarchaeota		[148,154]
*Candidatus* Woesearchaeota			[153]
**Domain Bacteria**			
Abawacabacteria	*Candidatus* Abawacaibacteriota		[66]
*Candidatus* Absconditabacteria	*Candidatus* Absconditibacteriota		[73]
*Candidatus* Adlerbacteria	*Candidatus* Adleribacteriota		[65]
*Candidatus* Amesbacteria	*Candidatus* Amesiibacteriota	*Candidatus* Amesibacteriota	[65]
*Candidatus* Aminanaerobia	*Candidatus* Aminanaerobiota		[155]
*Candidatus* Andersenbacteria	*Candidatus* Anderseniibacteriota	*Candidatus* Andersenibacteriota	[66]
*Candidatus* Aureabacteria	*Candidatus* Aurifodinibacteriota		[156]
*Candidatus* Azambacteria	*Candidatus* Azamiibacteriota	*Candidatus* Azamibacteriota	[65]
*Candidatus* Beckwithbacteria	*Candidatus* Beckwithiibacteriota	*Candidatus* Beckwithibacteriota	[65]
*Candidatus* Berkelbacteria	*Candidatus* Berkeliibacteriota	*Candidatus* Berkelibacteriota	[77]
*Candidatus* Blackallbacteria	*Candidatus* Blackalliibacteriota	*Candidatus* Blackallibacteriota	[75]
*Candidatus* Blackburnbacteria	*Candidatus* Blackburniibacteriota	*Candidatus* Blackburnibacteriota	[66]
*Candidatus* Brennerbacteria	*Candidatus* Brenneribacteriota		[66]
*Candidatus* Brownbacteria	*Candidatus* Browniibacteriota	*Candidatus* Brownibacteriota	[157]
*Candidatus* Buchananbacteria	*Candidatus* Buchananiibacteriota	*Candidatus* Buchananibacteriota	[66]
*Candidatus* Campbellbacteria	*Candidatus* Campbelliibacteriota	*Candidatus* Campbellibacteriota	[65]
*Candidatus* Chisholmbacteria	*Candidatus* Chisholmiibacteriota	*Candidatus* Chisholmibacteriota	[66]
*Candidatus* Coatesbacteria	*Candidatus* Coatesiibacteriota	*Candidatus* Coatesibacteriota	[66]
*Candidatus* Collierbacteria	*Candidatus* Collieribacteriota		[65]
*Candidatus* Colwellbacteria	*Candidatus* Colwelliibacteriota	*Candidatus* Colwellibacteriota	[66]
*Candidatus* Curtissbacteria	*Candidatus* Curtissiibacteriota	*Candidatus* Curtissibacteriota	[65]
*Candidatus* Dadabacteria	*Candidatus* Dadaibacteriota		[73]
*Candidatus* Daviesbacteria	*Candidatus* Daviesiibacteriota	*Candidatus* Daviesibacteriota	[65]
*Candidatus* Delongbacteria	*Candidatus* Delongiibacteriota	*Candidatus* Delongibacteriota	[66]
*Candidatus* Delphibacteria	*Candidatus* Delphinibacteriota		[70]
*Candidatus* Desantisbacteria	*Candidatus* Desantisiibacteriota	*Candidatus* Desantisibacteriota	[75]
*Candidatus* Dojkabacteria	*Candidatus* Dojkaibacteriota		[78]
*Candidatus* Doudnabacteria	*Candidatus* Doudnaibacteriota		[66]
*Candidatus* Edwardsbacteria	*Candidatus* Edwardsiibacteriota	*Candidatus* Edwardsibacteriota	[66]
*Candidatus* Eisenbacteria	*Candidatus* Eiseniibacteriota	*Candidatus* Eisenibacteriota	[66]
*Candidatus* Falkowbacteria	*Candidatus* Falkowiibacteriota	*Candidatus* Falkowibacteriota	[65]
*Candidatus* Fertabacteria	*Candidatus* Fallacibacteriota		[70]
*Candidatus* Firestonebacteria	*Candidatus* Firestoneibacteriota		[66]
*Candidatus* Fischerbacteria	*Candidatus* Fischeribacteriota		[66]
*Candidatus* Fraserbacteria	*Candidatus* Fraseribacteriota		[66]
*Candidatus* Genascibacteria	*Candidatus* Genasciibacteriota	*Candidatus* Genascibacteriota	[71]
*Candidatus* Giovannonibacteria	*Candidatus* Giovannoniibacteriota	*Candidatus* Giovannonibacteriota	[65]
*Candidatus* Glassbacteria	*Candidatus* Glassiibacteriota	*Candidatus* Glassibacteriota	[66]
*Candidatus* Goldbacteria	*Candidatus* Goldiibacteriota	*Candidatus* Goldibacteriota	[72]
*Candidatus* Gottesmanbacteria	*Candidatus* Gottesmaniibacteriota	*Candidatus* Gottesmanibacteriota	[65]
*Candidatus* Gribaldobacteria	*Candidatus* Gribaldonibacteriota		[75]
*Candidatus* Handelsmanbacteria	*Candidatus* Handelsmaniibacteriota	*Candidatus* Handelsmanibacteriota	[66]
*Candidatus* Harrisonbacteria	*Candidatus* Harrisoniibacteriota	*Candidatus* Harrisonibacteriota	[66]
*Candidatus* Hugbacteria	*Candidatus* Hugiibacteriota	*Candidatus* Hugibacteriota	[157]
*Candidatus* Jacksonbacteria	*Candidatus* Jacksoniibacteriota	*Candidatus* Jacksonibacteriota	[66]
*Candidatus* Jorgensenbacteria	*Candidatus* Joergenseniibacteriota	*Candidatus* Joergensenibacteriota	[65]
*Candidatus* Kaiserbacteria	*Candidatus* Kaiseribacteriota		[65]
*Candidatus* Katanobacteria	*Candidatus* Katanibacteriota		[73]
*Candidatus* Kerfeldbacteria	*Candidatus* Kerfeldiibacteriota	*Candidatus* Kerfeldibacteriota	[66]
*Candidatus* Komeilibacteria	*Candidatus* Komeiliibacteriota	*Candidatus* Komeilibacteriota	[66]
*Candidatus* Kuenenbacteria	*Candidatus* Kueneniibacteriota	*Candidatus* Kuenenibacteriota	[65]
*Candidatus* Levybacteria	*Candidatus* Levyibacteriota		[65]
*Candidatus* Lindowbacteria	*Candidatus* Lindowiibacteriota	*Candidatus* Lindowibacteriota	[66]
*Candidatus* Liptonbacteria	*Candidatus* Liptoniibacteriota	*Candidatus* Liptonibacteriota	[66]
*Candidatus* Lloydbacteria	*Candidatus* Lloydiibacteriota	*Candidatus* Lloydibacteriota	[66]
*Candidatus* Magasanikbacteria	*Candidatus* Magasanikiibacteriota	*Candidatus* Magasanikibacteriota	[65]
*Candidatus* Margulisbacteria	*Candidatus* Margulisiibacteriota	*Candidatus* Margulisibacteriota	[66]
*Candidatus* Melainabacteria	*Candidatus* Melainobacteriota		[69]
*Candidatus* Moisslbacteria	*Candidatus* Moissliibacteriota	*Candidatus* Moisslibacteriota	[76]
*Candidatus* Montesolbacteria	*Candidatus* Montesoliibacteriota	*Candidatus* Montesolibacteriota	[71]
*Candidatus* Moranbacteria	*Candidatus* Moraniibacteriota	*Candidatus* Moranibacteriota	[65]
*Candidatus* Nealsonbacteria	*Candidatus* Nealsoniibacteriota	*Candidatus* Nealsonibacteriota	[66]
*Candidatus* Niyogibacteria	*Candidatus* Niyogiibacteriota	*Candidatus* Niyogibacteriota	[66]
*Candidatus* Nomurabacteria	*Candidatus* Nomuraibacteriota		[65]
*Candidatus* Peregrinibacteria	*Candidatus* Peregrinibacteriota		[53,65]
*Candidatus* Poribacteria	*Candidatus* Poribacteriota		[158]
*Candidatus* Portnoybacteria	*Candidatus* Portnoyibacteriota		[66]
*Candidatus* Ratteibacteria	*Candidatus* Ratteibacteriota		[76]
*Candidatus* Raymondbacteria	*Candidatus* Raymondiibacteriota	*Candidatus* Raymondibacteriota	[66]
*Candidatus* Roizmanbacteria	*Candidatus* Roizmaniibacteriota	*Candidatus* Roizmanibacteriota	[65]
*Candidatus* Rokubacteria	*Candidatus* Rokuibacteriota		[73]
*Candidatus* Ryanbacteria	*Candidatus* Ryaniibacteriota	*Candidatus* Ryanibacteriota	[66]
*Candidatus* Saganbacteria	*Candidatus* Saganiibacteriota	*Candidatus* Saganibacteriota	[75]
*Candidatus* Schekmanbacteria	*Candidatus* Schekmaniibacteriota	*Candidatus* Schekmanibacteriota	[66]
*Candidatus* Shapirobacteria	*Candidatus* Shapironibacteriota		[65]
*Candidatus* Spechtbacteria	*Candidatus* Spechtiibacteriota	*Candidatus* Spechtibacteriota	[66]
*Candidatus* Stahlbacteria	*Candidatus* Stahliibacteriota	*Candidatus* Stahlibacteriota	[159]
*Candidatus* Staskawiczbacteria	*Candidatus* Staskawicziibacteriota	*Candidatus* Staskawiczibacteriota	[66]
*Candidatus* Sungbacteria	*Candidatus* Sungiibacteriota	*Candidatus* Sungibacteriota	[66]
*Candidatus* Tagabacteria	*Candidatus* Tagaibacteriota		[66]
*Candidatus* Taylorbacteria	*Candidatus* Tayloriibacteriota	*Candidatus* Tayloribacteriota	[66]
*Candidatus* Tectomicrobia	*Candidatus* Tectimicrobiota		[160]
*Candidatus* Terrybacteria	*Candidatus* Terryibacteriota		[66]
*Candidatus* Torokbacteria	*Candidatus* Torokiibacteriota	*Candidatus* Torokibacteriota	[76]
*Candidatus* Uhrbacteria	*Candidatus* Uhriibacteriota	*Candidatus* Uhribacteriota	[65]
*Candidatus* Veblenbacteria	*Candidatus* Vebleniibacteriota	*Candidatus* Veblenibacteriota	[66]
*Candidatus* Vogelbacteria	*Candidatus* Vogeliibacteriota	*Candidatus* Vogelibacteriota	[66]
*Candidatus* Wallbacteria	*Candidatus* Walliibacteriota	*Candidatus* Wallibacteriota	[66]
*Candidatus* Wildermuthbacteria	*Candidatus* Wildermuthiibacteriota	*Candidatus* Wildermuthibacteriota	[66]
*Candidatus* Woesebacteria	*Candidatus* Woeseibacteriota		[65]
*Candidatus* Wolfebacteria	*Candidatus* Wolfeibacteriota		[65]
*Candidatus* Woykebacteria	*Candidatus* Woykeibacteriota		[66]
*Candidatus* Yanofskybacteria	*Candidatus* Yanofskyibacteriota		[65]
*Candidatus* Yonathbacteria	*Candidatus* Yonathiibacteriota	*Candidatus* Yonathibacteriota	[66]
*Candidatus* Zambryskibacteria	*Candidatus* Zambryskiibacteriota	*Candidatus* Zambryskibacteriota	[66]
*Candidatus* Zixibacteria	*Candidatus* Zixiibacteriota	*Candidatus* Zixibacteriota	[67]

## Uncultured names

For nearly 30 years, uncultured prokaryotic taxa have been afforded a provisional status termed *Candidatus* (abbreviated to *Ca*.) [82], Given that most prokaryotic species have not yet—or probably never will—be cultured, it is not surprising the number of phyla with *Candidatus* names now vastly exceeds the number with validly published names. As part of an initiative to bring all *Candidatus* names in line with practice for validly published names, in 2023 Oren and Göker published a list of 170 names for *Candidatus* phyla, emended to satisfy recently agreed rules for naming prokaryotic phyla. In one case they noted that a *Candidatus* phylum previously named *Ca*. Atribacteria had lost *Candidatus* status on valid publication of a type genus name and had earned the validly published name *Atribacterota*.

For 69 phyla (Table 2), where a type genus had already been suggested, emendations generally led to minor changes to the previously used name (e.g. changing *Ca*. Aerophobetes to *Ca*. Aerophobota) and/or to a phylum name built in a predictable fashion from the type genus, e.g. *Ca*. Baldrarchaeota built from *Ca*. Baldrarchaeum). However, some emended names were not obviously related to previously published names, e.g. *Ca*. Dependentiae becomes *Ca*. Babelota; *Ca*. Thermoplasmatota becomes *Ca*. Poseidoniota.

In a provocative step, Oren and Göker suggested a set of well-formed names ending in -ota for over 100 *Candidatus* phyla, mostly those described by Banfield and colleagues, where no type genus had been designated. Here they reverse engineered the published phylum names to create *Candidatus* names for yet-to-be-described type genera (Table 3) that they used to generate their proposed phylum names. For example, they created the name for the non-existent type genus *Ca*. Falkowiibacterium to create the name *Ca*. Falkowiibacteriota for the phylum previously named *Ca*. Falkowbacteria. Unfortunately, they added an ugly and unnecessary double ‘ii’ to the ends of personal names, when, as I have argued elsewhere, a single ‘i’, e.g. as in *Ca*. Falkowibacteriota provides a more agreeable outcome [83]. It remains to be seen whether anyone will apply the proposed genus names to well-circumscribed biological entities, particularly as most of the associated phyla are not recognized at this rank in commonly used taxonomies.

## Phyla within a genome taxonomy

In 2018, Parks, Hugenholtz and their colleagues launched a standardized bacterial taxonomy with an associated database (the GTDB), based on concatenated protein phylogenies from single-copy genes from publicly available genome sequences [84]. They confronted the long-standing problems that result when uniform sequence thresholds are used to define prokaryotic taxa. Bearing in mind that as long ago as 1980, Woese and colleagues flagged mycoplasmas as showing anomalously rapid evolution [13], Parks *et al*. highlighted the genus *Mycoplasma* as a particular problem, as it shows sufficient within-genus diversity to represent two phyla on the basis of a 16S rRNA gene sequence identity threshold of 75%. To address this issue, they normalized higher ranks according to relative evolutionary divergence (RED), with extant taxa assigned a RED score of 1, the last common ancestor assigned a score of 0 and internal nodes assigned interpolated values in line with lineage-specific rates of evolution. Taxonomic rank normalization based on RED led in some cases to lumping previously named phyla together, while in other cases phyla were split.

Cases of lumping included the CPR, which was assigned to a single phylum, the *Patescibacteria,* adopting the name from what was previously designated a superphylum. Similarly, the phyla *Chlorobi* and *Ignavibacteriae* were lumped together as classes within the *Bacteroidetes*, while they followed Woese in demoting the phylum *Tenericutes,* home to the genus *Mycoplasma*, to a class within the *Firmicutes.*

The most prominent case of splitting within GTDB occurred with the *Proteobacteria,* which was split into six phyla—a process formally described in two later publications [85,86]. Some previously named phyla already split with the silva database were recorded as split within GTDB and marked with alphabetical suffixes, e.g. *Spirochaeta_A* as a lineage split from the phylum Spirochaeta. The authors questioned whether the *Firmicutes* (now called *Bacillota*) should be considered a stable coherent phylum, but at first retained it as a phylum-level lineage. However, in future releases, they split the group into nine phyla. Six additional phyla were also split with alphabetical suffixes: the *Bdellovibrionota*, the *Desulfobacterota*, the *Gemmatimonadota*, the *Myxococcota*, the *Nitrospinota* and the *Nitrospirota*. Unfortunately, GTDB fails to grant taxa with alphabetical suffixes the full status of phyla or give them well-formed Latin names in absence of solid evidence that they represent monophyletic groups. One hopes that this deficit can be rectified in the near future, particularly as most of these phyla-with-suffixes are home to cultured potential type genera (e.g. *Bacillota_A* could easily be renamed *Clostridiota* or *Bacillota_C* could be renamed *Selenomonadota*).

A more problematic issue with nomenclature arises because GTDB provides hierarchical sequence-based classifications for many prokaryotic candidate phyla that are identified by alphanumeric placeholder labels which are hard to remember and are easily confused (e.g. p__JAGLYR01 and p_JAGLTZ01). To address this issue, with colleagues I have published well-formed Latinate names with arbitrary derivations for over a hundred unnamed prokaryotic phyla and associated type genera within GTDB (Table 4) [87,88]. These arbitrary names have already seen use in research publications [89,92] and are likely to provide handy signposts to new functional and phylogenetic diversity.

**Table 4.** Candidate phyla with arbitrary names (as of November 2023)

*Candidatus* name	Synonyms	Genomes	G+C content (%)	Genome size (MBp)	Isolation sources	Authority for name	References
**Domain Archaea**							
*Candidatus* Acigarchota	JAADDD01	1	0.54	45.66	Deep-sea hydrothermal sulphide chimney	[88]	
*Candidatus* Axalarchota	EX4484-52	23	0.50–1.45	27.95–44.32	Deep-sea sediments, hydrothermal vents, methane seeps, marine sediments, groundwater, terrestrial mud volcano	[87]	
*Candidatus* Iduparchota	SpSt-1190	7	0.56–1.14	35.69–63.06	Groundwater, hydrothermal vents, hot springs	[87]	[161]
*Candidatus* Oferarchota	B1Sed10-29;	17	0.34–1.33	34.37–55.97	Cold saline springs, seep sediments, marine hydrothermal sediments, freshwater sediments, groundwater	[87]	
*Candidatus* Omefarchota	JACRDV01	1	1.54	36.46	Groundwater	[88]	
**Domain Bacteria**							
*Candidatus* Acidulodesulfobacteriota*	SZUA-79	70	0.88–3.04	28.23–43.58	Multiple acidic and marine environments, including acid mine drainage and a hydrothermal chimney	[87]	
*Candidatus* Afabiota	CG2-30-53-67	10	2.18–3.11	51.99–59.02	Diverse environments, including coastal, groundwater and hydrothermal samples	[87]	
*Candidatus* Afuciota	JAGLYR01	1	3.4	60.32	Deep-sea sediment	[88]	
*Candidatus* Asafiota	UBP6	32	1.69–3.59	28.61–60.78	A range of environments: sulphidic cave, freshwater, anaerobic sludge, hypersaline lake sediment, rumen	[87]	
*Candidatus* Axaliota	KSB1	76	2.22–10.27	37.25–61.79	Mix of aquatic environments: hydrothermal vents, chimneys, freshwater and bioreactors	[87]	[102,159, 162,168]
*Candidatus* Axiviota	JAGLTZ01	1	1.99	57.91–57.91	Deep-sea sediment	[88]	
*Candidatus* Bimafiota	UBP7	6	0.83–1.39	35.63–52.53	Saline and marine water environments, including plankton and suspended particulate matter	[87]	
*Candidatus* Binidiota	TA06	5	2.32–3	48.30–60.62	Deep-sea and Mariana trench sediments, along with methane-rich estuary sediments	[87]	[163,169,174]
*Candidatus* Bobupiota	JAJRZV01	1	2.45	47.78	Active black smoker, hydrothermal vent environment named DFF12	[88]	
*Candidatus* Busufiota	T1Sed10-126	2	2.15–2.2	46.12–48.13	Hypersaline soda lake sediment	[87]	
*Candidatus* Cenuriota	4572–55	2	4.09–4.18	46.29–48.47	Deep-sea hydrothermal vent sediments; iron-rich hot spring	[87]	
*Candidatus* Cobisiota	SLNR01	1	2.07	44.52	Hypersaline soda lake environment	[87]	
*Candidatus* Coxosiota	RBG-13-61-14	3	3.65–4.74	55.33–61.15	Freshwater and subsurface sediments	[87]	
*Candidatus* Cunadiota	DRYD01	2	1.39–1.4	30.21–32.84	Alkaline hot spring environment with brown biofilm	[87]	
*Candidatus* Cutipiota	UBA2233	2	1.8–1.89	55.75–56.11	Industrial environments: oil-contaminated soil and a tailings pond	[87]	
*Candidatus* Cuxufiota	B130-G9	3	2.27–3.43	53.92–56.18	Marine hydrothermal and deep-sea vent sediments alongside freshwater sediment	[87]	
*Candidatus* Dalofiota	JACPSX01	1	2.9	59.60	Groundwater	[87]	
*Candidatus* Debefiota	AABM5-125-2; Candidatus Electryoneota 4	43	1.23–4.91	41.91–66.09	Various aquatic and subsurface environments, including aquifers and bioreactors	[87]	[102,175,177]
*Candidatus* Dobariota	UBA6262	10	1.33–1.99	33.54–49.66	Marine and deep-sea environments, along with pelagic sediment and wastewater	[87]	[178]
*Candidatus* Dufaniota	JAHJDO01	2	1.35–2.68	37.91–38.14	Deep-sea sediment	[87]	
*Candidatus* Dufopiota	VGIX01	3	2.19–2.75	65.83–70.34	Microbial mats from a hypersaline environment and a freshwater lake	[87]	
*Candidatus* Dumaciota	RUG730	3	1.09–2.15	25.46–33.34	Marine sediment and unspecified environment, alongside cattle rumen	[87]	
*Candidatus* Dunuliota	UBP13	2	2.18–2.25	48.89–49.01	Mud	[87]	
*Candidatus* Dupeciota	SZUA-182	8	1.87–3.83	40.07–50.46	Marine hydrothermal sulphide and freshwater sediments, alongside hot springs	[87]	
*Candidatus* Efretiota	JAAXVQ01	5	2.59–3.59	44.61–56.05	Various aquatic environments: seep sediment culture, deep-sea, fracture fluid, polymer biofilm, freshwater sediment	[88]	
*Candidatus* Enediota	CSP1-3	30	1.49–4.43	61.91–71.86	Groundwater environments, sediment depths, freshwater lakes, grassland and an ammonia-oxidizing culture	[87]	34197190
*Candidatus* Esaciota	UBA10199	65	1.11–4.58	38.95–63.74	Freshwater and subsurface sediments	[87]	
*Candidatus* Falabiota	NPL-UPA2	5	0.91–1.81	43.74–47.50	Deep-sea, hydrothermal and groundwater environments, including spring water	[87]	[179,181]
*Candidatus* Femapiota	JABDJQ01	7	2.93–3.89	63.24–66.03	Marine and freshwater environments, black smoker, and fracture fluid	[87]	
*Candidatus* Figaniota	JACPQY01	2	3.29–4.72	60.76–64.61	Groundwater	[87]	
*Candidatus* Fitepiota	JAKLEM01	1	1.62	59.81–59.81	Anaerobic digester sludge	[88]	
*Candidatus* Fitomiota	UBA8481	3	2.33–3.21	42.22–57.58	Groundwater and an iron-rich hot spring	[87]	
*Candidatus* Fixabiota	JADJOY01	1	8.19	70.88	Activated sludge	[87]	
*Candidatus* Gesefiota	JAFGBW01	3	1.97–3.14	33.61–44.74	Freshwater and deep-sea sediments	[87]	
*Candidatus* Getofiota	CSSED10-310	9	2.57–9.2	43.37–65.89	Black smoker, freshwater sediment, and hypersaline soda lake	[87]	[182]
*Candidatus* Gulusiota	T1SED10-198M	1	2.55	42.37	Hypersaline soda lake	[87]	
*Candidatus* Hacexiota	JACIXR01	2	2.55–2.69	44.87–45.96	Oilfield environment at Shengli, Shandong province	[87]	
*Candidatus* Hicupiota	UBA3054	15	1.23–4.12	37.86–64.53	Rumen, organic waste digestion and hydrothermal vent	[87]	
*Candidatus* Honifiota	JAGOBX01	1	3.83	30.20	Wastewater	[87]	
*Candidatus* Hubebiota	JANLFM01	2	1.73–1.82	49.31–49.74	Enrichment cultures from a terrestrial mud volcano	[88]	
*Candidatus* Hudomiota	BMS3Abin14	12	1.54–2.86	51.36–63.39	Freshwater, marine hydrothermal sediment, and sub-seafloor sulphide environments	[87]	
*Candidatus* Hurebiota	JAFGND01	1	2.99	35.87	Freshwater sediment	[87]	
*Candidatus* Hydrothermota	WOR-3	121	0.89–4.21	29.93–68.33	Alkaline hot spring, freshwater sediment, hypersaline mats, and various aquatic environments	[87]	[163,165, 183]
*Candidatus* Ibidiota	UBA8248	28	1.47–4.37	35.26–70.66	Marine environments, including sponge tissue, brackish and saline waters with plankton, hydrothermal vents	[87]	[184]
*Candidatus* Ibociota	CAKKQC01	2	3.34–4.68	69.02–69.36	Unspecified and groundwater environments	[88]	
*Candidatus* Idrufiota	UBP14	12	1.29–3.98	40.81–54.45	Hydrothermal vent sediments, marine hydrothermal environments, and wastewater	[87]	
*Candidatus* Idupiota	CG03	23	0.72–2.53	33.39–51.43	Saline spring sediment, hypersaline soda lake, and freshwater sediments	[Bibr R87][87]	[185]
*Candidatus* Ifutiota	JAAXHH01	27	3.06–6.06	51.06–62.93	Freshwater, marine metagenomes, sponge tissue, and hypoxic seawater	[Bibr R87][87]	
*Candidatus* Inuciota	JAGRBM01	1	4.18	63.26	Sludge from Shatin wastewater treatment plant, collected over years	[Bibr R88][88]	
*Candidatus* Lagoxiota	UBP15	6	2.56–4.55	51.41–60.43	Black smoker, anaerobic digester sludge, and wastewater environments	[Bibr R87][87]	
*Candidatus* Lanaxiota	BS750m-G34	1	0.8	42.90	Brackish Black Sea water	[Bibr R87][87]	
*Candidatus* Locusiota	UBP18	5	0.78–1.31	44.25–47.46	Formation waters, hydrothermal vent sediment, and groundwater	[Bibr R87][87]	
*Candidatus* Luxamiota	FCPU426	6	2.23–4.1	46.69–57.09	Various soil and groundwater environments including permafrost	[88]	[178,186, 187]
*Candidatus* Macifiota	DUMJ01	3	1.76–2.37	37.65–39.79	Organic waste digestion and oilfield environments	[Bibr R87][87]	
*Candidatus* Megaciota	JAMCPX01	17	1–2.64	39.48–55.05	Acid mine drainage sediment	[88]	
*Candidatus* Moxediota	PUNC01	2	2.32–2.8	60.11–64.22	Hypersaline soda lake	[Bibr R87][87]	
*Candidatus* Naraxiota	JACRDZ01	3	2.3–3.08	30.63–49.03	Black smoker and groundwater environments	[Bibr R87][87]	
*Candidatus* Nasexiota	JAJYCY01	1	2.52	67.92	Acid mine drainage sediment	[Bibr R88][88]	
*Candidatus* Nerisiota	QNDG01	12	2.46–5.29	36.88–61.38	Freshwater, deep-sea sediment, hydrothermal vent sediment, Arctic lake environments	[Bibr R87][87]	
*Candidatus* Niteriota	4484–113	12	1.54–4.11	44.23–64.05	Aquatic bioreactor environments, including activated sludge from wastewater treatment	[Bibr R87][87]	[175]
*Candidatus* Ocupiota	CAIJMQ01	9	0.95–1.54	36.88–44.30	Hydrothermal chimney and pond	[Bibr R87][87]	
*Candidatus* Ogusiota	RBG-13-66-14	10	1.62–2.96	42.81–65.80	Deep-sea hydrothermal vents, microbial mats from hypersaline environment; subsurface sediments	[Bibr R87][87]	[175]
*Candidatus* Omexiota	UBA9089	19	1.09–3.11	34.17–44.14	Aquatic, sulfidic, groundwater, and alpine spring water environments	[Bibr R87][87]	
*Candidatus* Omubiota	CG2-30-70-394	11	2.5–3.06	69.49–70.52	Black smoker, filtered groundwater, and subsurface aquifer environments	[Bibr R87][87]	
*Candidatus* Ostegiota	UBA1439	3	2.41–2.59	68.67–68.79	Biodigester and organic waste digestor; anammox bioreactor	[Bibr R87][87]	
*Candidatus* Oviciota	JADFOP01	3	1.63–2.52	59.19–60.88	Sediments from the surface of the Mariana Trench	[Bibr R88][88]	
*Candidatus* Podoxiota	FEN-1099, also called *Ca*. Lernaellota	7	2.65–5.25	61.48–64.30	Bioreactor biomass, wastewater treatment sludge, and permafrost soil environments;	[Bibr R87][87]	
*Candidatus* Pudofiota	JACPUC01	1	4.46	66.75	Groundwater	[Bibr R87][87]	
*Candidatus* Puresiota	HKB111	4	1.24–2.12	38.39–39.10	Spring water	[Bibr R87][87]	
*Candidatus* Robemiota	CLD3	10	3.09–4.14	34.08–49.17	Various wastewater treatment and bioreactor environments	[Bibr R87][87]	
*Candidatus* Rosutiota	TA06_A	2	3.07–5.04	49.70–52.83	Estuary sediment environment rich in sulphate and methane	[Bibr R87][87]	
*Candidatus* Rudufiota	JACQOV01	1	1.9	49.33	Groundwater	[Bibr R87][87]	
*Candidatus* Saxiciota	JACPWU01	4	2.23–2.51	41.48–44.37	Groundwater	[Bibr R87][87]	
*Candidatus* Sifixiota	J088	1	2.83	62.40	Iron-rich hot spring	[Bibr R87][87]	
*Candidatus* Soduniota	UBA6266	5	1.47–2.26	39.39–44.92	Marine, anammox reactor, and wastewater treatment environments	[Bibr R87][87]	
*Candidatus* Sogexiota	UBP17	10	3.37–8.39	29.92–68.33	Marine and freshwater environments, including ammonox reactor	[Bibr R87][87]	
*Candidatus* Sonubiota	SM23-31	3	2.48–2.82	33.68–42.01	Deep-sea hydrothermal vent and Mariana trench sediments; sulphate-methane transition zone estuary sediments	[Bibr R87][87]	[102]
*Candidatus* Sucudiota	BS750m-G25	1	2.51	46.40	Brackish Black Sea water	[Bibr R87][87]	
*Candidatus* Tefagiota	JAGOEH01	1	3.58	48.74	Wastewater	[Bibr R87][87]	
*Candidatus* Tibeniota	GCA-001730085	2	1.66–1.91	34.69–43.24	Mariana Trench surface sediment and ovicells	[Bibr R87][87]	
*Candidatus* Tufoliota	ARS69	3	1.68–3.82	65.60–69.71	Wastewater treatment bioreactor and marine sample	[Bibr R87][87]	[89]
*Candidatus* Tusixiota	UBP4	2	3.36–3.56	43.46–43.49	Wastewater	[Bibr R87][87]	
*Candidatus* Tuxefiota	JABMQX01	2	4.06–4.48	56.28–65.34	Freshwater sediment and High-Arctic meromictic lake	[Bibr R87][87]	
*Candidatus* Ucifiota	DYJV01	1	2.51	32.56	Biofilm from sulphidic cave	[Bibr R88][88]	
*Candidatus* Udisiota	JdFR-76	15	1.43–5.37	39.26–51.84	Deep-sea hydrothermal vent and Mariana Trench sediments	[Bibr R87][87]	[176]
*Candidatus* Urusiota (also called *Candidatus* Hinthialibacterota)	OLB16	21	3.39–7.54	49.66–65.03	Freshwater sediment and various geothermal environments	[Bibr R87][87]	[188,189]
*Candidatus* Usiniota	DTU030	15	1.22–2.88	47.09–63.03	Anaerobic digestion and a range of bioreactor environments for organic waste processing	[Bibr R87][87]	
*Candidatus* Usuriota	SAR324	147	1.26–8.98	32.37–69.55	Hypoxic seawater, subsurface aquifer, coral colonies, hydrothermal plumes, and marine environment	[Bibr R87][87]	[190,191]
*Candidatus* Uvuciota	AUK180	3	2.45–3.12	32.88–43.18	Deep-sea and marine hydrothermal sediments	[Bibr R88][88]	

*aA non-arbitrary name built from a named type genus *Candidatus* Acidulodesulfobacterium.

## Unexplored biodiversity

While a few of the candidate phyla have been studied in some detail, most represent ‘known unknowns’ in that, we know they exist, but remain largely ignorant of their genomic or phenotypic diversity, ecological content or environmental range. Metabolic and functional reconstructions from genomes available from uncharacterized phyla represents an easy win for those interested in pushing the boundaries of prokaryotic biodiversity. A brief glimpse at isolation sources for genomes from phyla recently demarcated in GTDB highlights a wide range of aqueous and terrestrial environments (Table 4). However, at least three—*Ca*. Dumaciota, *Ca*. Hicupiota and *Ca*. Asafiota—are found in the rumen, suggesting that we might yet find additional new phyla in host-associated microbiomes.

Even a brief glimpse at phylogenies suggests phylogenetic diversity within prokaryotic phyla is decidedly non-uniform. According to GTDB, among the 181 bacterial phyla, there are currently five with ≥10 000 genome sequences, 16 with ≥1000 genomes, 50 with ≥100 genomes and 98 with ≥10 genomes. Alarmingly, for over a quarter of phyla, we have three or fewer genome sequences (Table 4). Many of these differences in genome representation probably represent sampling bias, with attention focused on contexts of immediate medical, industrial or ecological importance. This bias could be addressed by deliberating seeking out additional members of genome-poor phyla.

However, it is also clear that some phyla are richer in daughter lineages than others. For example, according to GTDB, the phylum *Planctomycetota* harbours 28 classes but less than 3000 genomes, while *Cyanobacteriota* is home to over 4000 genomes, but only three classes. Does the paucity of classes within *Cyanobacteriota* reflect a highly streamlined set of ecological roles adopted at an early stage? Does the diversity of classes in *Planctomycetota* indicate functional versatility and niche partitioning driven by highly variable ecological contexts? Elucidation of the evolutionary and ecological forces driving early cladogenesis within phyla remains an interesting challenge for future research.

## Do phyla still matter?

In traditional Linnaean taxonomy, the rank of phylum represents a defined level within a taxonomic hierarchy. However, when we switch to a phylogenetic approach, one can argue that it is arbitrary to privilege some clades as ranks and leave others unnamed and unclassified—a view championed by proponents of PhyloCode [93]. For example, if we assume that the Candidate Phyla Radiation (largely at least) represents a monophyletic group, does it matter whether we call it a clade, a phylum or a superphylum?

By contrast, careful analysis of distance metrics and rank-normalization by the GTDB team provides a strong argument for the enduring utility and reality of the rank of phylum in prokaryotic taxonomy [94]. A recent analysis from Göker and Oren [95] provides additional support in describing a strong log–linear relationship between number of taxa and rank, again suggesting that prokaryotic phyla represent meaningful categories rather than just arbitrary clades. Interestingly, my own observations suggest this log–linear relationship appears to be independent of any specific taxonomic view, with similar patterns seen when looking at ranks defined by the LPSN, GTDB and NCBI.

Another concern is that now we have hundreds of phyla—with so many names and properties that no one remember all of them—have they ceased to represent something fundamental? Interestingly, the analysis by Göker and Oren [95] suggests that there might be two missing ranks between the rank of phylum and the domains Bacteria and Archaea. More recently, Göker and Oren followed up on this finding to subdivide the domain *Bacteria* into the kingdoms *Bacillati*, *Fusobacteriati*, *Pseudomonadati* and *Thermotogati* and the domain *Archaea* into the kingdoms *Methanobacteriati*, *Nanobdellati* and *Thermoproteati*. While it remains to be seen whether these ranks above phyla are generally accepted by the scientific community (particularly given the problems of painting new names on to old taxa highlighted above), there is clear evidence from a number of studies for the existence of two major high-level clades generally called the *Terrabacteria* (or *Bacillati* according to Göker and Oren) and the *Gracilicutes* or *Hydrobacteria* (or *Pseudomonadati* according to Göker and Oren) [96,100].

Theoretical arguments aside, there are practical reasons why prokaryotic phyla still matter. For analyses of microbiomes, classification of microbial communities at phylum level remains a popular approach providing a handy, informative and standardized overview of a microbial community that allows comparative analyses spanning different hosts, environments, locations, or timescales. This is particularly pertinent in the clinical setting, where the ratio of *Bacteroidetes* to *Firmicutes* (or as we should say now, the ratio of *Bacteroidota* to *Bacillota*) in the gut has been cited in hundreds of papers and has been linked to the balance between health and disease, particularly in obesity [101]. However, one could also argue that the near-ubiquitous presentation of phylum-level data in visually appealing but scientifically shallow pie charts approach offers more aesthetic value than scientific insight, particularly as it is now possible to resolve communities down to the level of strains. In addition, as we noted earlier, ever since Woese, categorizing prokaryotes into phyla based on phylogenetic grounds leaves open the question of how far members of any given phylum share any meaningful ecological and functional traits.

## Future prospects

In closing, it is worth noting that many unresolved issues remain in the nomenclature, taxonomy and characterization of prokaryotic phyla (Table 5). There are numerous candidate phyla that lack well formed names or type genera. Remarkably there are still some candidate phyla known only from 16S rRNA sequences for which we have no genome sequences. In these cases, it may be worth re-exploring the environments, samples and sequences associated with the phyla to retrieve complete genome sequences. It is also worth attempting to reconciling 16S rRNA-based and genome-based phylogenies with phenotypes, as illustrated by a recent genomic characterization of the Candidate Division LCP-89 that revealed an atypical cell-wall structure [102].

**Table 5.** Unresolved issues in prokaryotic phylum nomenclature and classification

**Phylum**	Comments	Isolation sources	References
10bav-F6	silva phylum known only from 16S sequences	Marine sediments	[192]
AncK6	silva phylum known only from 16S sequences	Coral and benthic sediments	[193,194]
Apal-E12	silva phylum known only from 16S sequences	Deep sea sediment	[195]
BHI80-139	silva phylum known only from 16S sequences	Marine sediments	[192,196, 197]
CK-2C2-2	silva phylum known only from 16S sequences	Marine sediments; salty grassland	[197,198]
DTB120	silva phylum, could be renamed *Ca*. Ferristratota after genus *Ca*. Ferristratum, but no species named	Marine sediments; hydrothermal vent	[192,199, 200]
FW113	silva phylum known only from 16S sequences	Wastewater, deep sea sediment	[195,201]
GAL15	silva phylum known only from 16S sequences	Coral, subsurface strata	[193,202]
GN01	silva phylum known only from 16S sequences		[203]
JL-ETNP-Z39	NCBI candidate division known only from 16S sequences	Sea water	[204]
KD3-62	NCBI candidate division with genome sequences, but no named genera	Estuarine sediment	[163]
Lambdaproteobacteria	Candidate phylum name without type genus, not listed in IJSEM		[66]
LCP-89	Silva phylum with genome sequences, but no named genera	Anoxic spring, marine sediments	[102,197]
MAT-CR-M4-B07	silva phylum known only from 16S sequences	Amoebozoa, hypersaline microbial mat	[205,206]
Methylomirabilota	Published as a candidate phylum name but not yet listed in IJSEM; synonym candidate division NC10		[103]
Muproteobacteria	Candidate phylum name without type genus, not listed in IJSEM		[66]
NB1-j	silva phylum known only from 16S sequences		[207,208]
NKB15	silva phylum known only from 16S sequences	Marine sediments	[197]
OP6	NCBI candidate division known only from 16S sequences		[41]
OS-K	NCBI candidate division known only from 16S sequences	Spring, phreatic sinkhole, alpine tundra wet meadow soil, molybdenum mine	[38]
OP7	NCBI candidate division known only from 16S sequences		[41]
Patescibacteria	GTDB phylum with Ill-formed name and without type genus		[84]
PAUC34f	Silva phylum with genome sequences, but no named genera	Freshwater sediments, the terrestrial subsurface, dark ocean bacterioplankton	[209]
RCP2-54	silva phylum known only from 16S sequences	Marine sediments, forested wetland	[197,210]
Rs-K70	Silva phylum, but reported elsewhere as a deeply branching clade in the Deltaproteobacteria		[211]
Sva0485	Silva phylum, but reported elsewhere as an order in the Deltaproteobacteria		[212]
TX1A-33	silva phylum known only from 16S sequences	Cave sediments, saline soil	[213,214]
WOR-1	Silva phylum, but reported in GTDB as a class in the phylum Margulisbacteria		[163]
WOR-3	NCBI candidate division with genome sequences, but no named genera	Estuarine sediment	[163]
WS1	Silva phylum with genome sequences, but no named genera	Contaminated aquifer	[42,215]
WS2	Silva phylum with genome sequences, but no named genera, probably a clade within Patescibacteria	Marine sediments, anaerobic digester; contaminated aquifer	[42,197]
WS4	silva phylum known only from 16S sequences	Contaminated aquifer	[42]

The hunt for new bacterial phyla remains an open and promising line of research, fuelled by advances in sequencing and bioinformatics. However, whether the pace of discovery will level off soon remains unclear. On one hand, the growing sophistication of analytical tools and the increasing ease of obtaining metagenomic data suggest that many more discoveries await. On the other hand, diminishing returns may set in as the opportunity to sample truly novel environments levels off. However, it is clear that—with a backlog of 100 or more yet to be characterized—prokaryotic phyla will continue to provide work aplenty for microbiologists for decades to come.

## Abbreviations

Ca.: Candidatus
CPR: Candidate Phyla Radiation
GTDB: Genome Taxonomy Database
ICNP: International Code of Nomenclature of Prokaryotes
ICSP: International Committee on Systematics of Prokaryotes
LPSN: List of Prokaryotic Names with Standing in Nomenclature
MAGs: Metagenome-Assembled Genomes
NCBI: National Center for Biotechnology Information
RED: Relative Evolutionary Divergence
